# Development and validation of a rapid loop-mediated isothermal amplification assay for the detection of *Chrysomyxa* and characterization of *Chrysomyxa woroninii* overwintering on *Picea* in China

**DOI:** 10.1186/s43008-024-00157-6

**Published:** 2024-08-07

**Authors:** Wan Ting Yu, Xin Wang, Tan Yin, Clement Kin-Ming Tsui, Chong Juan You

**Affiliations:** 1 Beijing Key Laboratory for Forest Pest Control, Beijing Forestry University, Beijing, 100083 China; 2 Infectious Disease Research Laboratory, National Centre for Infectious Diseases, Tan Tock Seng Hospital, Singapore, 308433 Singapore; 3 Faculty of Medicine, University of British Columbia, Vancouver, V6T 1Z3 Canada

**Keywords:** DNA-based diagnostics, *Chrysomyxa*, LAMP, Spruce needle rust, Bud rust

## Abstract

*Chrysomyxa* rusts cause significant damage to spruce in both natural forests and plantations. Particularly, Three *Chrysomyxa* species, *Chrysomyxa deformans*, *Chrysomyxa qilianensis*, and *Chrysomyxa rhododendri*, listed as National Forest Dangerous Pests in China, have severely affected many economically and ecologically important spruce native species in China. Also, *Chrysomyxa arctostaphyli*, an important plant quarantine fungus, causes a damaging broom rust disease on spruce. Therefore, rapid, and efficient detection tools are urgently needed for proper rust disease detection and management. In this study, a sensitive, genus-specific loop-mediated isothermal amplification (LAMP) assay targeting the ITS-28S rRNA region was developed to detect the presence of *Chrysomyxa* in spruce needle and bud samples. After optimization and validation, the LAMP assay was found to be sensitive to detect as low as 5.2 fg/µL DNA, making it suitable for rapid on-site testing for rust infection. The assay was also specific to *Chrysomyxa* species, with no positive signals from other rust genus/species. The application of LAMP in the early detection of rust infections in spruce needles and buds was investigated, and spatial colonization profiles as well as the means of overwintering of *Chrysomyxa woroninii* in infected buds and branches were verified using the LAMP assay. This LAMP detection method will facilitate further studies on the characteristics of the life cycle and inoculation of other systemic rusts.

**Supplementary Information:**

The online version contains supplementary material available at 10.1186/s43008-024-00157-6.

## Introduction

Spruce rusts caused by *Chrysomyxa* Unger, are among the most damaging diseases of spruce in plantations and natural forests in the northern hemisphere, causing premature defoliation, growth reduction and in severe cases mortality, leading to important economic losses in the forestry (Crane et al. 2000; Cummins and Hiratsuka 1983; Feau et al. 2011; You et al. 2019; Wang et al. 2022). For instance, *Chrysomyxa deformans* (Diet.) Jacz., *Chrysomyxa qilianensis* Wang, Wu et Li, and *Chrysomyxa rhododendri* (DC.) de Bary, agents of spruce needle and cone rusts, have severely affected many economically and ecologically important spruce native species such as *Picea crassifolia* Kom. and *Picea purpurea* Mast, and these three species are listed as National Forest Dangerous Pests in China (Cao et al. 2017a, b; Wang et al. 2022). The spruce broom rust, caused by *Chrysomyxa arctostaphyli*, has also contributed to the decline of spruce. Predominantly found in North America, *C. arctostaphyli* is classified as a quarantine pathogen, and its introduction into China is prohibited due to its potential threat (Jeger et al. 2018; EPPO 2022a, b; http://dzs.customs.gov.cn/dzs/2746776/3699554/index.html). This pathogen could pose significant risks to spruce stands in Asia and Europe, where susceptible hosts and favorable climatic conditions are present (Ziller 1974; Jeger et al. 2018). The detection frequency of non-native invasive pathogens has significantly increased with the continued importation of spruce seedlings, highlighting the need for rapid and specific diagnostic tools to support the implementation of effective phytosanitary measures (Boutigny et al. 2013a).

Spruce rust damage can resemble that caused by spruce needle cast (*Lophodermium picea* (Fuckel) Höhn.) or other rust species. For instance, many *Chrysomyxa* species produce symptoms such as yellow to orange bands on current-year needles, similar to the symptoms of *Pucciniastrum goeppertianum* on *Abies* (Sickle 1977). Furthermore, infections by *Chrysomyxa pirolate* Wint., a well-known pathogen of spruce cone rusts, and the rust pathogen *Thekopsora areolate* (Fr.) Magnus also cause the similar type of symptoms, appearing as yellow spore masses on cone scales (Kaitera et al. 2009; Zhang et al. 2021). Microscopic examinations and conventional PCR of infected cones are required to distinguish the two from one another (Feau et al. 2011; Wang et al. 2022). To date, it is difficult to visually identify and detect the rust fungi in symptomatic plants as well as in asymptomatic tissues of diseased plants, due to the fact that most rust fungi are obligate biotrophs that may be propagated by asymptomatic plant material, resulting in a lack of sensitivity in visual inspections (Boutigny et al. 2013a; Lamarche et al. 2015; Bergeron et al. 2019).

Spruce bud rust, caused by *Chrysomyxa woroninii* Tranz*.*, is widely distributed in boreal forest of subalpine and far northern regions, including Canada, the United States, Russia, Japan, Switzerland and China (Savile 1950; Spaulding 1961; McBeath 1984; Wood and Heath 1986; He et al. 1995; Crane et al. 2000). This disease may retard growth of heavily infected seedlings in spruce regeneration areas, resulting in stunted shoot formation when buds and female cones are infected (McBeat 1981, 1984; Crane et al. 2000). *Chrysomyxa woroninii* is considered to be heteroecious, with a life cycle alternating between spermogonial and aecial stages on *Picea* and uredinial and telial stages on *Ledum*, as confirmed by Crane et al. (2000) through inoculation experiment. They indicated that *C. woroninii* has specific environmental requirements for survival, producing aecia only on two *Picea* trees with snow cover. However, McBeath (1984) suggested that the bud rust fungus is autoecious, with reinfection of spruce by aeciospores occurring on adjacent shoots later in the growing season. In China, spruce bud rust has been recorded on *P. koraiensis* Nakai. in Northeastern China. He et al. (1995) reported successful infection of Korean spruce two years after inoculation with *C. woroninii* aeciospores, but noted that the fungus was unable to sporulate normally on the field-collected *Ledum* or other *Rhododendron* plants, and details of their experiments were not provided. Recently, increased damage from spruce bud rust has been detected on *P. likiangensis* var. *Rubescens* in Sichuan Province, and *P. crassifolia* Kom. in Qinghai Province (southwestern and northwestern regions of China). However, the only possible alternate host *Ledum* spp. has been known to be absent from Western China. The lack of detailed inoculation experimental evidence for the aecial-telial hosts connection from Northeastern China, and the absence of the possible telial host *Ledum* spp. in Western China, has led to confusion about the life cycle of *C. woroninii* in China. Furthermore, understanding of the rust infection progress in *Picea* and the means of overwintering of the rust fungus in China is limited due to its complex life cycle. Studying the reproduction mode in rust fungi through inoculation experiments is time consuming and difficult, as the inoculation results often depend on the number of plants inoculated, their growth stage, and the source and amount of inoculum (Knott 1989; Pretorius et al. 2019). In contrast, molecular detection can simplify the connection between different life cycle stages via DNA sequences (Aime et al. 2017), and can be useful to investigate the colonization profiles of rust fungi in infected shoots or leaves (Hietala and Crossley 2006).

PCR and quantitative real-time PCR- based molecular methods are widely used in diagnosing tree rust diseases due to their efficiency, sensitivity, and ability to detect and identify rust fungi early (de la Bastide et al. 1995; Boutigny et al. 2013a, 2013b; Husson et al. 2013; Guinet et al. 2016; Feau et al. 2018). Rust-specific PCR enabled DNA to be detected in both symptomatic and asymptomatic parts of diseased plants, making it a valuable tool for studying the development of rust infections in inoculated plants. This method has been used to investigate the timing of rust infections and the movement of the fungus through the leaves, stems, or even the roots of infected plants (de la Bastide et al. 1995). Bergeron et al. (2019) developed species-and genus-specific real-time qPCR assays using the GEDI (Genome-enhanced detection and identification) approach, achieving 100% detection accuracy for pine and poplar rust diseases across different samples.

Loop-mediated isothermal amplification (LAMP) is an alternative technology to traditional PCR-based methods that is rapid, sensitive, efficient, and easily adaptable to the field settings with limited technical resources, which use four to six oligonucleotide primers and a DNA polymerase capable of synthesizing DNA at a constant temperature with strand displacement activity, thus eliminating the need for thermal cyclers (Notomi et al. 2000; Harrison et al. 2017; Myrholm et al. 2021). Various LAMP assays have been developed for point-of-care diagnosis of several forest pathogens, including *Heterobasidion irregulare*, *Dothistroma septosporum, Hymenoscyphus fraxineus* (Boonham et al. 2008; Harrison et al. 2017; Sillo et al. 2018; Myrholm et al. 2021). However, LAMP assays have only been applied to detect two rust species: *Cronartium ribicola* and *Phakopsora pachyrhizi* (Kozhar et al. 2023; Ouyang et al. 2023).

Currently, we have been investigating spruce rust diseases in China, focusing on the biology and taxonomy of, *Chrysomyxa*. In the paper, we first report the development of a LAMP-based assay for the rapid and accurate identification of *Chrysomyxa* species. Second, we evaluate its performance for detecting of *C. qilianensis* and *C. woroninii*, the most common spruce rust species in China that cause severe economic losses. Third, we utilize the LAMP assay to characterize the infection processes and the means of overwintering of *C. woroninii* on spruce in Western China, where increased damage has been observed on native spruce in recent years (Wang et al. 2022). This LAMP detection assay should greatly facilitate further studies on the biology of this and other systemic forest rusts.

## Materials and methods

### Sample collection, and DNA extraction

A nationwide collection of fresh rust specimens from target and non-target genera within the families *Coleosporiaceae*, *Pucciniastraceae*, and *Phakopsoraceae* has been deposited at the Mycological Herbarium, Museum of Beijing Forestry University, Beijing, China (BJFC). All specimens in our study were identified based on morphological evidence and phylogenetic analysis using a combined LSU and ITS2 rRNA sequence dataset, with the ITS4BRf/LR5 and ITS-Y3/ITS-Y4 primers (Vilgalys and Hester 1990; Vialle et al. 2009; Crane 2001; You et al. 2010, 2019; Feau et al. 2011; Cao et al. 2017a, b; Wang et al. 2022). Most of the rust samples on spruce were identified as belonging to ten *Chrysomyxa* species, which are widely distributed in China (Cao et al. 2017a, b; You et al. 2019). Host plants, locality of collection and samples numbers used in this study are listed in additional Table S1.

Genomic DNA was extracted from approximately 150–200 urediospores or aeciospores (15 to 20 μL in volume) scraped from a single uredinium or aecium using the QIAamp DNA Microbiome kit (Qiagen, Hilden, Germany) in accordance with the manufacturer’s instructions. Initial DNA concentrations were measured using a Qubit dsDNA HS Assay Kit (Invitrogen) and the NanoDrop 2000 Spectrophotometer (Thermo Fisher Scientific, Waltham, MA, USA). For comparison and to confirm the specificity of the proposed LAMP methods for *Chrysomyxa*, other spruce-associated filamentous fungi causing spruce needle cast, including *Rhizosphaera kalkhoffii* and *Lophodermium piceae*, were purchased from the China Forestry Culture Collection Center (CFCC). DNA from these pure cultures was isolated using the DNeasy Plant Mini Kit (Qiagen, Hilden, Germany), following the manufacturer’s protocol.

### DNA extraction from spruce materials

Spruce needles or buds suspected of *Chrysomyxa* infection, identified by microscopic examination showing yellow bands on needles and/or orange spore masses on buds, as well as visually healthy needles or buds, were selected for DNA extraction. About 0.025–0.03 g of needle and bud tissue was crushed to powder in liquid nitrogen using a glass rod inside a 1.5 ml microcentrifuge tube. Total DNA was then extracted using the DNeasy Plant Mini Kit (Qiagen, Hilden, Germany) in accordance with manufacturer’s instructions.

### Development of LAMP assay primers

In this study, the ITS2-28S rRNA region was selected as the target for our LAMP assay as it has been previously shown to distinguish *Chrysomyxa* from close relatives (Wang et al. 2022). The internal transcribed spacer 2 (ITS2) of the rRNA gene is particularly effective for discriminating most pine and spruce rust species, including *Chrysomyxa* and *Coleosporium*, because the ITS1 region often contains indels or paralogous copies that impede direct sequencing (Aime et al. 2017; McTaggart et al. 2018; Stewart et al. 2018; Heeger et al. 2019; Chen et al. 2022). The locations of rRNA gene regions (5.8S, ITS2, 28S) within representative *Chrysomyxa* sequences from our previous studies (You et al. 2019; Wang et al. 2022) and Feau et al. (2011), encompassing *Chrysomyxa* diversity in China and North America, were annotated by ITSx (Bengtsson-Palme et al. 2013). Sequences containing both ITS2 and at least 50 bp of the 28S region were retained (Chen et al. 2022). The consensus sequence of representative *Chrysomyxa* ITS2-28S gene sequences was then compared to corresponding portions of ITS2-28S gene sequences from related genera *Thekopsora* (MG787102.1-MG787138.1), *Coleosporium* (KY783662.1-KY783687.1; OK356493.1-OK356499.1) and *Pucciniastrum* (OL471658.1-OL471672.1; OL813493.1-OL813495.1; AF426226.1-AF426234. MT162683.1; MK488299.1; MK518915.1; MZ700104.1; MZ700229.1; OP339845.1) to identify *Chrysomyxa* genus-specific regions suitable for designing LAMP primers (Rebollar-Alviter et al 2011; Zhao et al. 2022). A set of LAMP primers targeting these specific regions of the *Chrysomyxa* was developed using Primer Explorer V5 software program (http://primerexplorer.jp). The specificity of the F3/B3 primer pair and the component sequences of the FIP and BIP primers (F1c and F2, and B1c and B2, respectively) (Additional file 1: Table S2) were assessed using Primer-BLAST.

### LAMP reaction and conditions

The LAMP assay was performed in a total volume of 25 µL consisting of ultra-pure water, 1.6 µM for primers FIP/BIP, 0.2 µM for primers F3/B3, 5 mM betaine, 1.4 mM dNTPs, 6 mM MgSO_4_, 8 U of Bst 3.0 DNA polymerase, 2.5 µL of 10 × Bst Reaction (final 1 ×), and 1 µL of template DNA (5.2 ng/µL). The optimal conditions of the LAMP assay were determined by running at different temperatures (45, 50, 55, 60, 65, 70, 75 and 80 °C) and reaction times (15, 30, 45, 60 and 75 min).

### LAMP detection with SYBR green I and real-time monitoring of the LAMP

The reaction results were directly detected via visual color changes observed with the naked eye in natural light by adding 0.75 µL of SYBR Green I dye mixture (Solarbio, Beijing) to the completed reaction. The dye was initially placed on the cover of the reaction tube before the reaction and mixed well after the reaction was completed. The solution changed from light orange to bright green in the presence of LAMP amplicons, implying that *Chrysomxya* DNA was detected, while a negative reaction remained light orange, indicating no amplification. Visualization of LAMP product was further confirmed via 1% agarose gel electrophoresis.

The LAMP assay was also monitored by Applied Biosystems™ ABI 7500 Fast Real-Time PCR System (ThermoFisher, USA). The real-time LAMP reaction was performed using the same sets of test samples and primers as described above, with the addition of 0.8 µL of SYBR Green I dye mixture (Solarbio, Beijing). Amplification was performed at 63 °C for 50 min, followed by annealing from 98 to 80 °C with ramping at 0.05 °C per second. The results were analyzed in terms of Tp values (the amplification time taken to generate a positive result, indicated by the peak of the fluorescence derivative) and the annealing temperatures (Ta) values (the temperature at which the fluorescence derivative reaches its maximum during the annealing process) (Harrison et al. 2017; Singh et al. 2020).

### Specificity of the LAMP assay

To investigate the specificity of the assay, DNA was extracted from a total of one hundred and twenty samples of ten representative *Chrysomyxa* species collected from various geographic locations in China over different years (Additional file 1: Table S1). Non-target rust DNA samples from related rust fungal genera (three from *Coleosporium* sp., one from *Phakopsora* sp., one from *Pucciniastrum* sp., one from *Thekopsora* sp., one from *Uredinopsis* sp., and DNA samples from spruce needle cast (*R. kalkhoffii* and *L. piceae*), were used as DNA templates (5.2 ng/µL). Specificity tests were repeated three times. Negative controls contained nuclease-free water in place of template DNA.

### Sensitivity of the LAMP and real-time PCR assays

The sensitivity of the LAMP assay was determined using tenfold serial dilutions of gDNA extracted from *C. qilianensis*, which is the most common spruce rust species in China. Initial DNA concentrations of three samples were measured using Qubit dsDNA HS Assay Kit (Invitrogen) and standardized to 5.2 ng/µL. Serial dilutions were then prepared, ranging from 5.2 × 10^−1^ ng/µL to 5.2 × 10^−7^ ng/µL. Each dilution was tested in duplicate, and this was repeated thrice.

The sensitivity of the Real-time PCR assay was also evaluated and compared with that of the LAMP assay, by testing a tenfold dilution series of *C. qilianensis* gDNA from 5.2 ng/µL to 5.2 × 10^−3^ ng/µL. Rust-enhanced ITS2 primers Rust2inv (5′-GATGAAGAACACAGTGAAA-3′) and ITS4(5′-CAGGAGACTTGTACACGGTCCAG-3′) (Aime 2006; Chen et al. 2022) were carried out in the real-time PCR. The reaction was performed in a 20 µL consisting of 10 µL of 2 × TB Green Premix Ex Taq II (Tli RNaseH Plus), 0.4 µL ROX Reference Dye II (50X), 0.4 µM each primer, 2µL template DNA and 6 μL of nuclease‐free water. The Applied Biosystems™ 7500 Fast Real-Time PCR System (Thermo Fisher, USA) was used for amplification and fluorescence measurement at each temperature step and cycle during the reaction. Thermal cycling conditions consisted of an initial 30 s at 95 °C, followed by 40 cycles at 95 °C for 5 s, and annealing at 60 °C for 34 s.

### Application of LAMP assay for early detection of *Chrysomyxa* from infected spruce

To further confirm the efficiency of LAMP assays for the early detection of *Chrysomyxa* from spruce needles, three putatively infected needles and symptomatic needles from naturally infected spruce by *C. qilianensis* were collected in July 2021 from Jiangla Forest Farm in Qinghai Province, respectively. Three healthy current-year needles were collected from trees without any signs or symptoms of rust infection, from where a field location with no known occurrence of spruce rust. Each tree was marked so that symptom expression could be noted during August to September of 2021. The LAMP reactions were repeated twice as described above.

### LAMP detection of *C. woroninii* from spruce buds

To investigate the spatial colonization profiles of *C. woroninii* in spruce buds and needles, heavily infected Chuanxi spruce (*Picea likiangensis var. rubescens* Rehder & E. H. Wilson) with bud rust from Mugecuo Scenic Area in Kangding, Sichuan Province, were collected between July and August 2021–2022. Infected terminal buds, potential uninfected subtending buds, visually healthy needles located 3–4 cm below the terminal buds, and internodes from 10 spruce trees, were sampled spatially by taking 0.02–0.03 g of samples from the edges of the diseased areas. DNA extraction and LAMP assays were performed twice as described above.

To further clarify the means of overwintering of *C. woroninii* in China, infected Qinghai spruce (*Picea crassifolia* Kom.) from Maixiu Forest Farm in Qinghai Province, were tagged and monitored from May 2021 to October 2022. Symptoms of bud rust disease, their time of appearance, and their development were recorded. A total of 10 nursery seedlings infected with bud rust were collected from the same site in October 2021, and in March, May, and July 2022, respectively. Before conducting LAMP assays, DNA extracts from different spruce tissue samples were checked with real-time PCR for all stages of the rust fungus. Terminal buds, subtending buds, and internodes were then detected using the previously described LAMP assays.

## Results

### LAMP primers

**Fig. 1:**
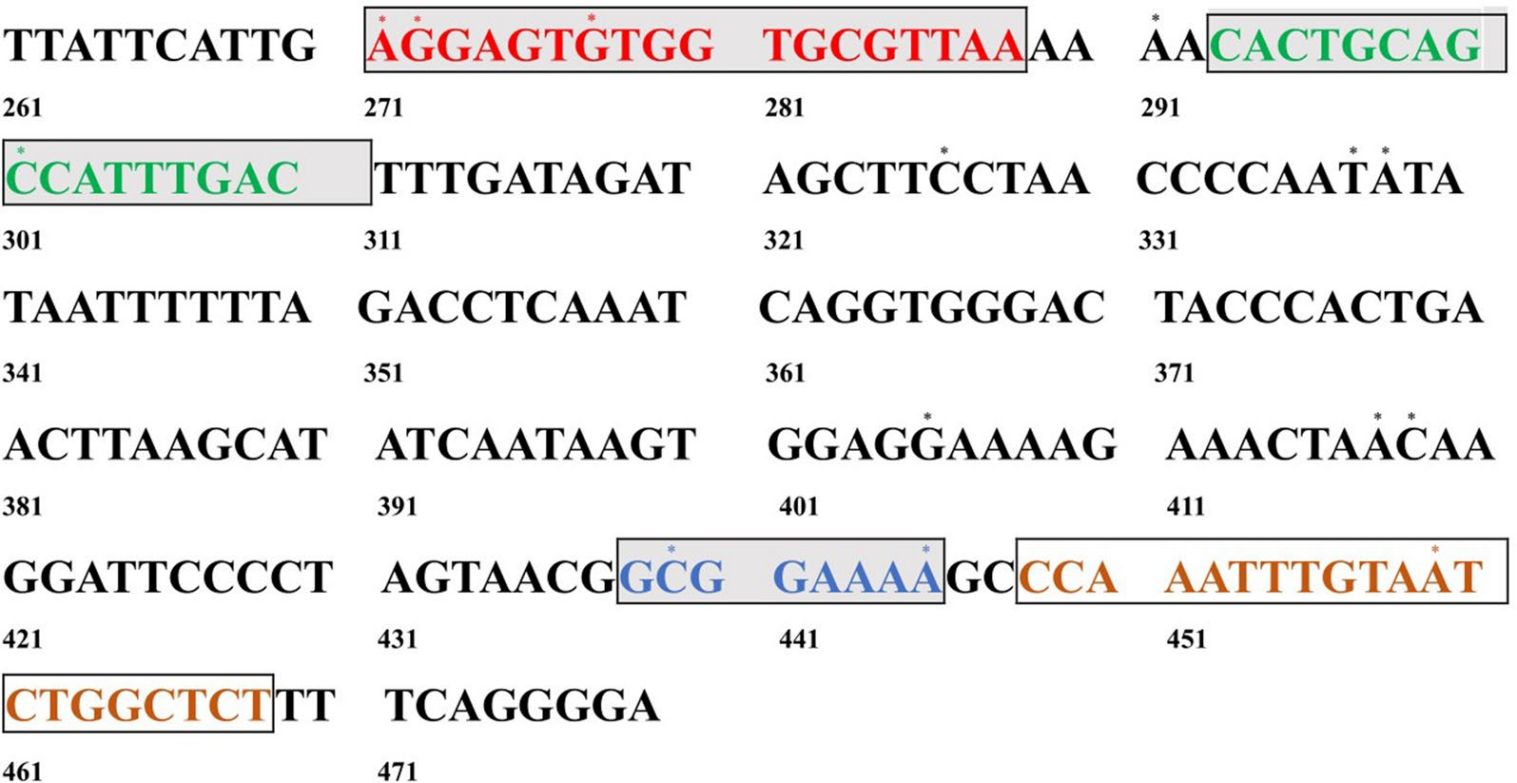
Location of primers for the LAMP assay for *Chrysomyxa* species. Primer FIP is a combination of F1c and F2, while primer BIP is the combination of B1c and B2. Differences from *Chrysomyxa* are noted by the * symbol

The consensus sequence of representative *Chrysomyxa* ITS2-28S rRNA sequences was compared to sequence data from related rust genera to identify *Chrysomyxa* genus-specific regions for LAMP primer design. Thirteen sets of LAMP primers, comprising two outer (F3 and B3) and two inner (FIP and BIP) primers, were designed based on the conserved and unique *Chrysomyxa* ITS2-28S gene sequence (Additional file 1: Table S2). Ten *Chrysomyxa* species widely distributed in China were selected for the practical application test of the genus-specific LAMP primer groups. Optimal primers were chosen for their ability to positively and specifically amplify the ten targeted *Chrysomyxa* species (Additional file 1: Table S3; Additional file 2: Figure S1). Finally, a set of four primers, exhibiting high genus-specificity and sensitivity and shorter detection times, were selected for further study. The sequence, location, and target details of the designed optimal primers are given in Table 1 and Fig. 1. The LAMP reaction mixture was then incubated at an optimal temperature of 64 °C for 45 min, followed by incubation at 80 °C for 10 min (Additional file 2: Figure S2).

**Table 1:** LAMP primers specific to the ITS2-28 s rRNA gene of *Chrysomyxa*

Primer name	Sequence 5′-3′
2-F3	AGGAGTGTGGTGCGTTAA
2-B3	AGAGCCAGATTACAAATTTGG
2-FIP	TCCCACCTGATTTGAGGTCTAAAAA-CACTGCAGCCATTTGACT
2-BIP	ACCCACTGAACTTAAGCATATCAA-TTTTCCCTCTTCACTCGC
2-F2	CACTGCAGCCATTTGACT
2-F1c	TCCCACCTGATTTGAGGTCTAAAAA
2-B2	TTTTCCCTCTTCACTCGC
2-B1c	ACCCACTGAACTTAAGCATATCAA

### Specificity and sensitivity of the LAMP

**Fig. 2:**
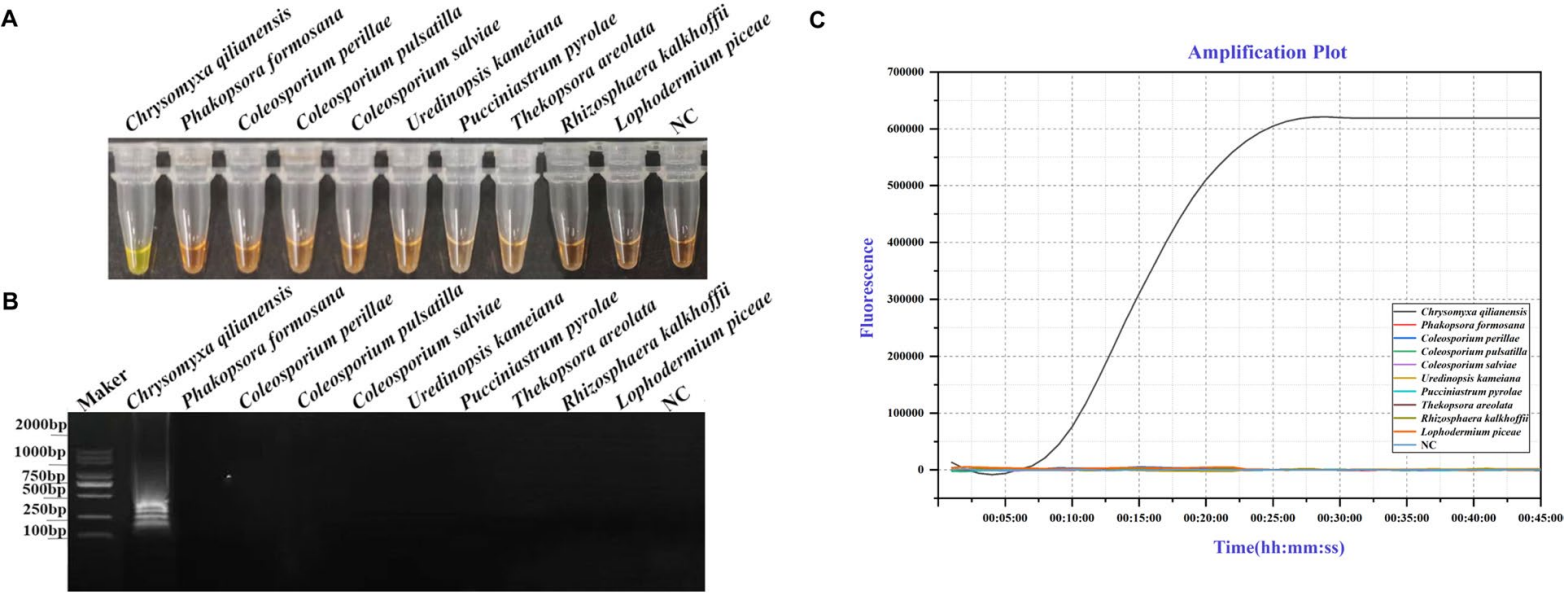
Specificity of the *Chrysomyxa* specific LAMP assay. **A** Visual. Green color indicated the detection of DNA fragments specific to *Chrysomyxa* species; orange color indicated the lack of detection. **B** On gel. Bands indicated the presence of LAMP amplicons. Lack of bands indicated no amplification. **C** On real-time qPCR instrument, amplification signals above the threshold indicated the detection of *Chrysomyxa* DNA; no PCR amplicon was detected in negative control (NC, ddH2O)

The specificity of the LAMP primers was evaluated with DNA extracted from *Chrysomyxa*, and other non-targeted fungi. In visual LAMP, positive amplification, as a change in color from orange to green, was detected only in *Chrysomyxa* samples (Fig. 2A; Additional file 1: Table S4). Also, a ladder-like pattern was observed via gel electrophoresis of the LAMP amplified products (Fig. 2B). The specificity of real-time LAMP assays was further confirmed by the fluorescence increase along with the amplification time. Successful detection occurred only in *Chrysomyxa* samples, with no amplification in non-target DNA and negative controls. *Chrysomyxa* samples tested positive in every replicated test and could be detected within 25–30 min (Fig. 2C). Consequently, the newly developed LAMP assay employing the optimal primers and reaction conditions demonstrated high genus-specificity for detecting *Chrysomyxa*.

**Fig. 3:**
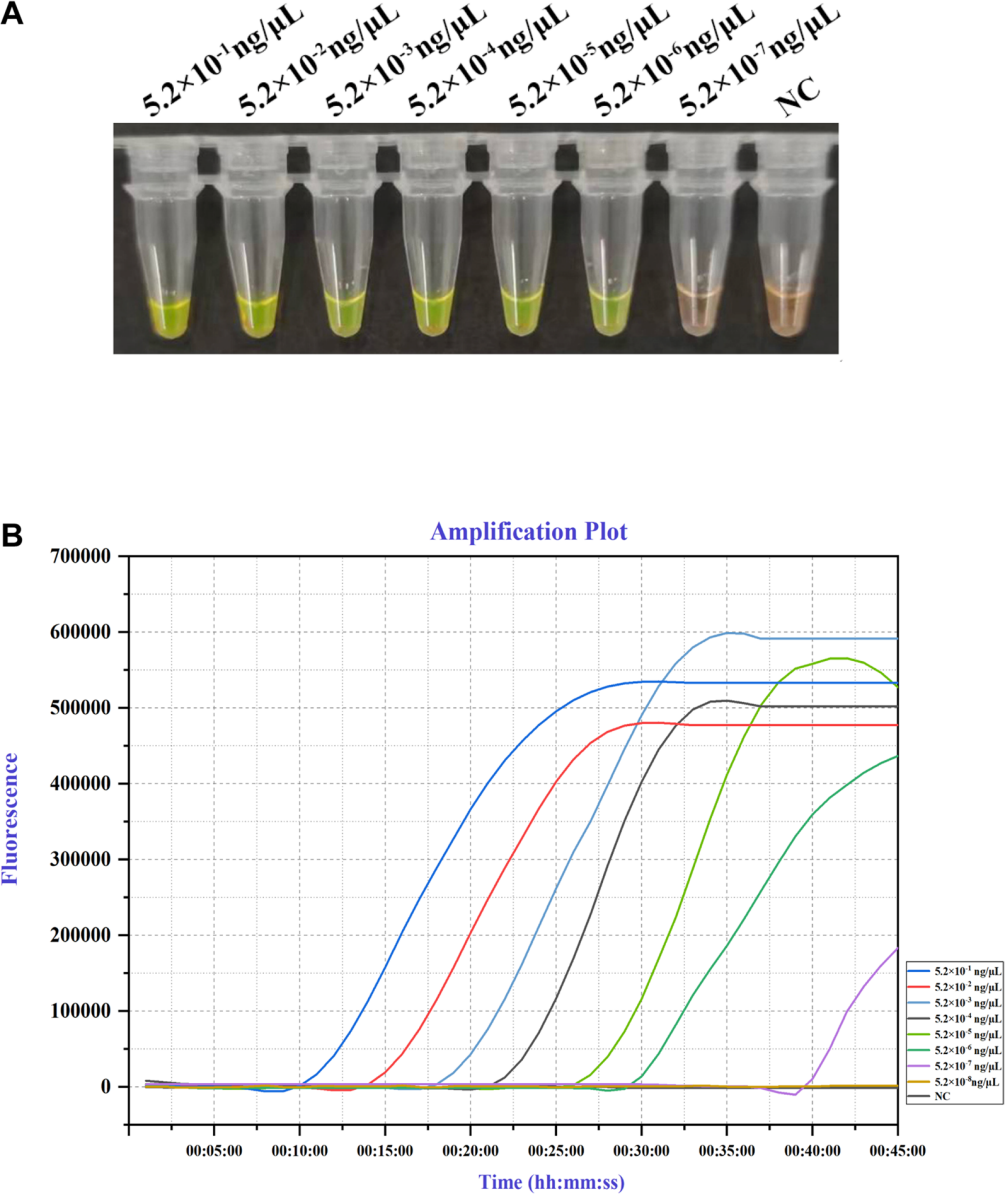
Sensitivity of LAMP assays. **A** In visual LAMP, green color indicated the detection of *Chrysomyxa* DNA. **B** In Real-time LAMP assay, amplification curves above threshold indicated the amplification of PCR fragments. *NC* Negative control (ddH2O)

The sensitivity of LAMP assay was determined by preparing a tenfold dilution series from 5.2 × 10^−1^ ng to 5.2 × 10^−7^ ng/µL of *C. qilianensis* DNA extracted from aeciospores. The change in color from orange to green was detected in the test samples in DNA concentration up to 5.2 × 10^–6^ ng/µL, where all replicate reactions (2/2) yielded positive results in every experiment (Fig. 3; Additional file 2: Figure S3). Increased dilutions substantially delayed the timing of signal above threshold, and no amplification was observed for dilutions over 5.2 × 10^−6^ ng/μL before 45 min (Fig. 3B). For comparison, real-time PCR using rust-specific ITS2 primers was performed on a tenfold dilution series of *C. qilianensis* DNA from 5.2 ng/µL to 5.2 × 10^−3^ ng/µL. The detection limit of our real-time PCR assay was approximately 5.2 × 10^–2^ ng/µL (Additional file 2: Figure S4). This indicates that the LAMP assay could detect *C. qilianensis* DNA concentration 10,000 times higher than that of real-time PCR assay; LAMP assay had greater sensitivity than regular qPCR.

### Early detection of *Chrysomyxa* from infected spruce using LAMP assay

**Fig. 4:**
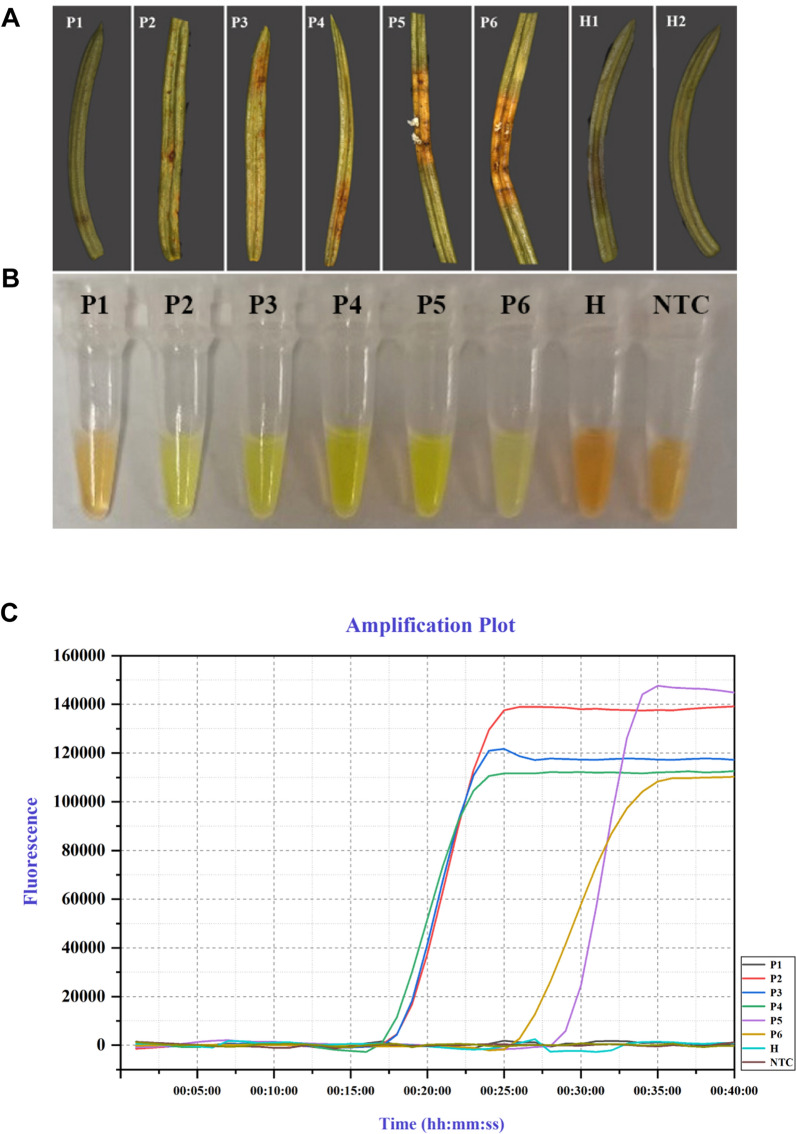
Early detection of *Chrysomyxa* from infected spruce by the LAMP assay. **A** Needle infection in different stages. (P1–P2: needles with yellow to orange spots; P3-P4: needles with yellow to orange bands; P5–P6: severely infected brown needles with powdery, orange spores; H1-H2: Healthy current-year needles; NTC: Non-template control) **B** Visual. Green color indicated the detection of *Chrysomyxa* DNA in samples of P2, P3, P4, P5 and P6 and no change in color was detected in the rest of the samples. **C** In Real-time LAMP assay, positive amplification was detected as real-time amplification curves

The application of the LAMP assay for early detection of *C. qilianensis* in spruce tissue was assessed using suspected infected needles and symptomatic current-year needles. Successful LAMP detection (both color change and real-time PCR) were obtained in samples suspected to be infected (P2: needles with yellow to orange spots; P3-P4: yellow to orange bands on current-year needles) and in symptomatic needles (P5-P6: severely infected brown needles with powdery, orange spores) (Fig. 4A). The positive reaction indicated that rust colonization had occurred in these spruce needles even before typical symptoms had fully developed. However, the suspected infected needles (P1) from a diseased spruce tree showed a negative amplification product. These samples were retested with both the LAMP assay and real-time PCR, and no amplification was detected in the repeated experiments. Furthermore, none of the healthy current-year needles (H1–H2) (without any signs or symptoms of rust infection and from where without known occurrences of spruce needle rust) showed any positive reaction, indicating the absence of rust fungi in these healthy needles. These results demonstrated that our LAMP assay enabled rust fungi to be detected in symptomatic needles as well as at early infectious stage in spruce needles.

### LAMP detection of *C. woroninii* from spruce buds

**Fig. 5:**
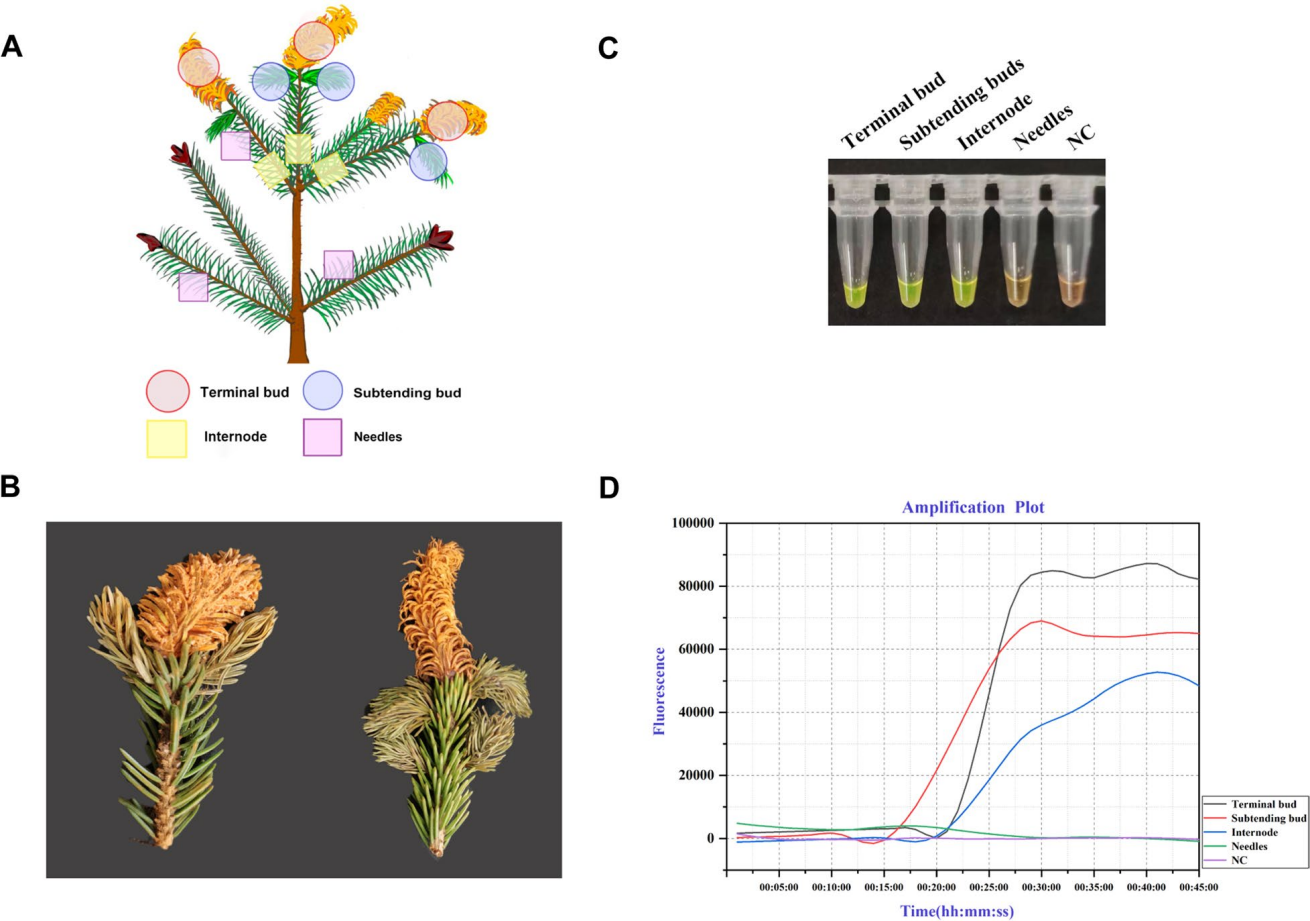
Spatial colonization of spruce bud rust pathogen *C. woroninii* reflected by LAMP. **A**: Illustrates the infected terminal buds, subtending buds and internodes on Chuanxi Spruce. **B**: Symptoms of bud rust on Chuanxi Spruce. **C**: LAMP assays. Positive amplifications (Green color) were observed in samples from terminal buds, subtending buds, and internode. **D**: In real-time qPCR instrument, amplification curves above threshold indicated the amplification of PCR fragments. *NC* Negative control (ddH2O)

To investigate the spatial colonization profiles of the spruce bud rust pathogen *C. woroninii* in buds and needles, heavily infected Chuanxi spruce samples were collected and tested by our developed LAMP assay. Positive amplifications were observed in all repeated reactions of DNA extracts from infected terminal buds, appearing uninfected subtending buds, and the internodes (Fig. 5A, B), whereas no amplification was observed in the needles below terminal buds for 3–4 cm, or in the non-template control (Fig. 5C, D). Our results confirmed that *C. woroninii* infects terminal buds and adjacent subtending buds rather than the needles.

**Fig. 6:**
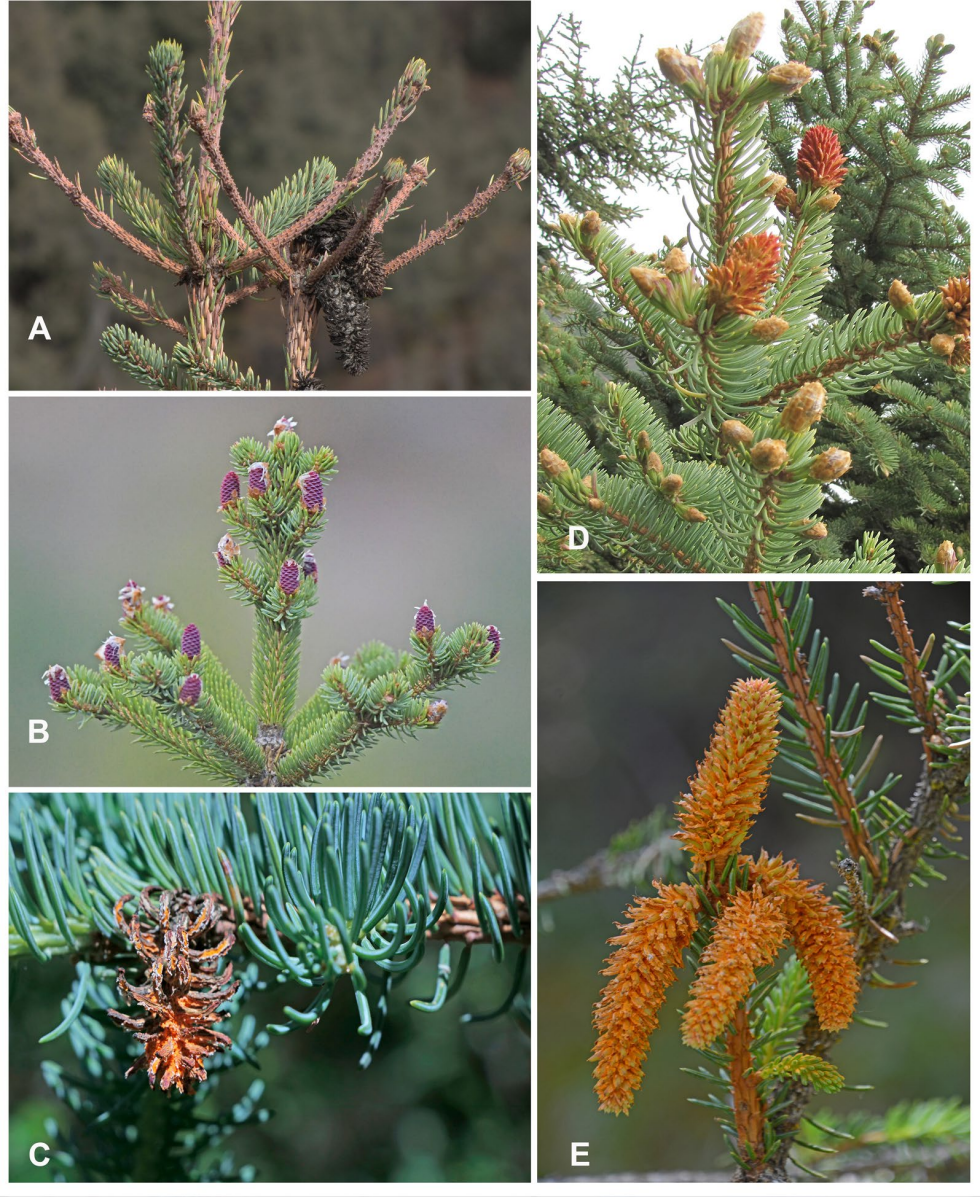
Typical symptoms of *C. woroninii* infection in Qinghai spruce at different periods. (**A** October 2021, **B** May 2022, **C** August 2022, **D** June 2022, **E** July 2022)

Infected Qinghai Spruce were tagged, and the development of bud rust disease was recorded from May 2021 to October 2022. The infection period lasted for two to three months. On 20 May 2021, several small red infected buds were visible, and yellow tiny spots developed on needles of last year’s shoots. On 25 June 2021, many severely infected buds were observed, producing masses of bright oranges aeciospores that were dispersed by wind. On 26 July 2021, infected buds were dying up and turning black, with adjacent buds slightly discolored and twisted; on 1st October 2021, infected buds had turned black, and lateral branches were discolored and gray. On 23 March 2022, buds infected last year were blackened (Fig. 6).

**Fig. 7:**
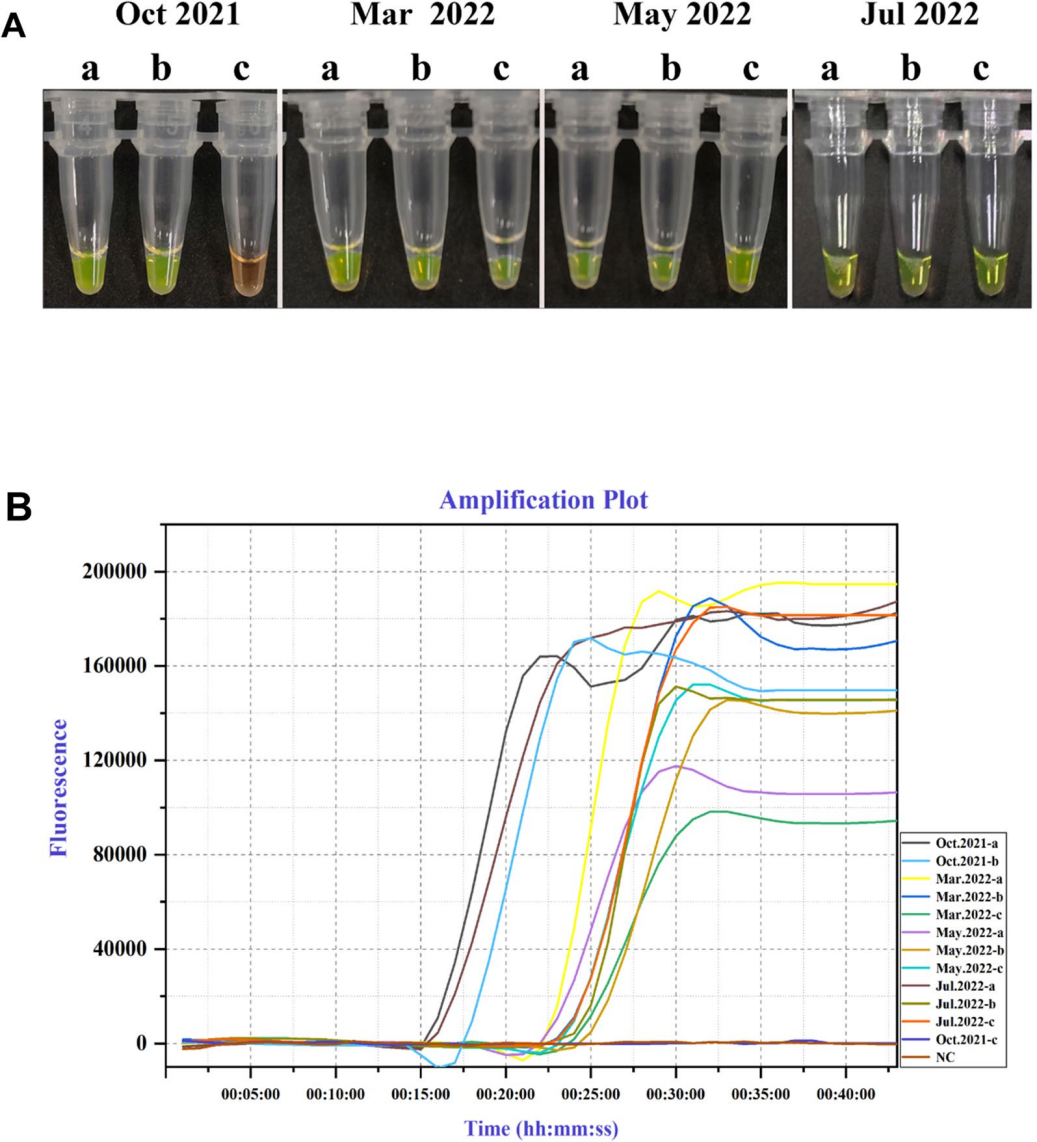
The detection of infected spruce samples collected from four different periods by LAMP. (a terminal buds, b: subtending buds, c: internode,) **A** LAMP assays, positive amplifications were observed except for spruce samples from internode collected from Oct 2021. **B** In real-time qPCR instrument, amplification curves above threshold indicated the amplification of PCR fragments. *NC* Negative control (ddH2O)

LAMP assay and real-time PCR were conducted to further clarify the means of overwintering of *C. woroninii* on Qinghai spruce in China. A total of 10 nursery seedlings from the same site were sampled in four different months (October 2021, March 2022, May 2022, July 2022). Terminal buds, subtending buds, and internodes for each sample group were tested using the developed LAMP assays. In samples collected in October 2021, successful visual detections were obtained in terminal buds and subtending buds, whereas no amplification was obtained in the internodes. In the other three cases (samples collected in March 2022, May 2022, July 2022), positive amplifications were detected when DNA from all three tissues of infected spruce (a: terminal buds, b: subtending buds, c: the internodes) (Fig. 7A). The results were confirmed by real-time PCR (Fig. 7B), which suggested that *C. woroninii* can overwinter within terminal and subtending buds. In spring and summer, hyphae may grow into or proliferate within the succulent tissue of the expanding shoot tips during the growing season when infection takes place and then into the buds.

## Discussion

The *Chrysomyxa* genus-specific LAMP assay developed in this study is based on the ITS2-28S gene, a locus previously shown to effectively distinguish *Chrysomyxa* from close relatives (McTaggart et al. 2018; Heeger et al. 2019; Chen et al. 2022). Our results further confirm the suitability of this locus as a diagnostic marker for *Chrysomyxa*. The LAMP assay successfully detected representative of ten *Chrysomyxa* spp. widely distributed in China. Additionally, our findings indicate that the LAMP assay can differentiate spruce rust damage from spruce needle cast (*Lophodermium picea*) and other rust species that produce similar symptoms during infections.

Our LAMP assay can detect *Chrysomyxa* DNA at concentrations as low as 5.2 fg/µL, demonstrating greater sensitivity compared to the real-time PCR method. Boutigny et al. (2013a) developed a 28S-based real-time PCR assay for detecting two *Melampsora medusae formae speciales* on infected poplar leaves, and it enabled the reproducible detection of two *M. medusae* urediniospore in a mixture of 2 mg of urediniospores (ca 800 000 urediniospores) from other *Melampsora* species. Additionally, to detect pine and poplar leaf rust pathogens from environmental samples, species- and genus-specific real-time PCR assays, targeting unique regions based on whole genome comparison, were reported, with sensitivity up to a single rust spore (Bergeron et al. 2019).

The application of the developed LAMP assays for early detection of symptomatic spruce needles was verified, and our data yielded satisfactory results compared to traditional identification methods. In China, spruce needle rust caused by *C. qilianensis* has become increasingly significant in recent years, particularly after an outbreak on Qinghai spruce (Wang et al. 1987, 2022). *Chrysomyxa qilianensis* is considered to be demicyclic, a reduced life cycle that lacks the uredinial state (Wang et al. 1987). It affects current-year needles and overwinters with hyphae in the infected needles. LAMP reaction data indicated that current-year spruce needles were colonized by *C. qilianensis* even before typical symptoms had developed. In one example (Fig. 4), P1 LAMP yielded a negative reaction when DNA from needle samples at the early infectious stage was tested. This could be be due to the tested needle materials being rust-free or having rust DNA concentrations below the detectable limit. But the second possibility was minimized by repeated high-sensitive LAMP tests. The dilution series indicated that our LAMP assay could detect as low as 5.2 fg/µL of DNA, making it unlikely that infected needles with rust hyphae or spores would go undetected. During August to September 2021, adjacent needles sampled from the same tree did not show signs of disease. In addition, it may be attributable to non-uniform pathogen distribution within infected tissue (Ioos et al. 2009; Gherghel et al. 2014). Gherghel et al. (2014) found that more rust fungal DNA could be recovered from the leading edge of symptomatic lesions and from non-symptomatic material above the lesions than from other parts of the plant.

In our study, we clarified the yearly disease cycle of spruce bud rust in Western China through a field survey. The period of infection of *C. woroninii* on spruce tree extends from May to late July (Crane et al. 2000). However, our LAMP results revealed positive amplifications from bud samples of asymptomatic spruce collected in March, indicating that some unopened buds were infected, suggesting that the infection can occurred at a very early stage of bud development.

Symptoms of infection by *C. woroninii* became clearly visible in mid-to-late May, when the buds started to unfold. Small, papillate, yellowish spermogonia were found exclusively at the apical region of the needles. Generally, *C. woroninii* primarily attacked apical buds but occasionally affected lateral buds as well (Fig. 5), as previously indicated by McBeath (1984) and Crane et al. (2000). Our visual LAMP results confirmed the infection of lateral bud (Fig. 5). During the summer from late June through July, all needles on shoots arising from rust-infected buds turned bright yellow and became severely stunted, though some lateral shoots displayed non-typical symptoms of bud rust infection (Fig. 5). Our LAMP assay can also detect minute amounts of *C. woroninii* in asymptomatic parts of diseased plants, including subtending buds and internodes. However, no amplification was observed in needles 3-4 cm below the terminal buds, indicating these needles are the most unlikely infection sites on spruce when infection takes place. In Autumn (October), infected buds turned dark brown, and the infected shoots gradually became dehydrated. LAMP analysis showed successful visual detections in terminal and subtending buds collected in Oct, whereas no amplification was observed in the internodes. It is possible that rust hyphae grow into or proliferate within the succulent tissue of the expanding shoot tips during the growing season and then into the winter buds. This growth continues until the buds become dormant in autumn, allowing the rust fungus to establish and overwinter in these buds (Heide 1974; Owens and Molder 1976; Crane et al. 2000). For Qinghai Spruce, the first cell divisions occurred within the buds in late March, marking the end of dormancy. Flushing occurred in early July, and shoot elongation was completed by late August. So the infection occurs when overwintered buds have just opened, with minimal expansion of the shoot axis between the needles from September until the following spring when the buds break dormancy (Owens and Molder 1976; Zhu et al. 2021).

In our study, we used LAMP to investigate the spatial colonization profiles of *C. woroninii* in infected buds and branches, as well as the asymptomatic parts of diseased spruce. The results indicated that the rust fungus overwinters with hyphae in infected buds and that its hyphae were also detected below the buds in the pith and cortex of the stem during the growing season (from March to July). However, none hyphae were found within the twigs or shoot apex below the buds during the dormancy season (from September to February). The autoecism life cycle of *C. woroninii* from Western China, is not well documented in our study. However, our developed LAMP assay will be valuable for optimizing inoculation methods in future research. Early recognition of infected spruce could eliminate the need to wait for symptoms appeared in the following year. This technique can be used to monitor the development of rust infections in inoculated plants, including the timing and spread of the fungus through the infected plants.

## Conclusions

In summary, the developed LAMP method targeting ITS2-28S gene was sensitive and specific for detecting *Chrysomyxa* in spruce needles and buds. Our results indicate that this LAMP assay enables the early detection of rust infections in spruce when they are asymptomatic. Additionally, the LAMP assay appeared to be useful for investigating the spatial colonization profiles of spruce bud rust pathogen *C. woroninii* and studying its means of overwintering in China. In future study, the LAMP-based diagnostic method should be optimized for in-field detection of tree rusts and it should be adopted in clarifying the life cycle of tree rust fungi through inoculation experiments.

### Supplementary Information

Additional file 1 Table S1. Fungal samples used for the LAMP assay development. Table S2. Primers used for the detection of *Chrysomyxa* using LAMP assays. Table S3. Detection of thirteen sets of *Chrysomyxa* genus-specific LAMP primers using visual LAMP and gel electrophoresis. 1. Clr: Visual inspection of the LAMP results. The positive reaction indicated by color change from light orange to light green (Green circles). The negative reaction remained light orange (Orange circles). 2. Ele: Agarose gel electrophoresis of LAMP products. Positive result (+). Negative result (−). Table S4. Comparison of analytical specificity and sensitivity of the LAMP assays and real-time PCR.

Additional file 2 Figure. S1. Detection of the optimal LAMP primers for *Chrysomyxa* species. A. Visual: green color indicated the detection of DNA fragments specific to *Chrysomyxa* species; orange color indicates the lack of detection. B: In real-time qPCR equipment: amplification signals above the threshold indicated the detection of *Chrysomyxa* DNA; no PCR amplicon was detected in negative control (NC, ddH2O). Figure. S2. Optimization of temperature and time of LAMP reaction. Figure. S3. Sensitivity of LAMP assays assessed by gel electrophoresis analysis (Concentration of diluted DNA template from 5.2 × 10^−1^ to 5.2 × 10^−7^ ng/μL.). Figure. S4. Sensitivity of Real-time PCR assay in detecting *Chrysomyxa*. Positive results for concentrations from 5.2 ng/μL to 5.2 × 10^−2^ng/μL. Negative results for 5.2 × 10^−3^ and negative control (ddH2O).

## Data Availability

Data of this study are included in the article or Supplementary Materials.
