# Supplementary material for: Development and validation of a rapid loop-mediated isothermal amplification assay for the detection of Chrysomyxa and characterization of Chrysomyxa woroninii overwintering on Picea in China
Source: IMA Fungus. 2024 Aug 7;15:23. doi: 10.1186/s43008-024-00157-6 (PMC11304928; doi:10.1186/s43008-024-00157-6)
Supplement: Supplementary file 1 — Additional file 1 Table S1. Fungal samples used for the LAMP assay development. Table S2. Primers used for the detection of Chrysomyxa using LAMP assays. Table S3. Detection of thirteen sets of Chrysomyxa genus-specific LAMP primers using visual LAMP and gel electrophoresis. 1. Clr: Visual inspection of the LAMP results. The positive reaction indicated by color change from light orange to light green (Green circles). The negative reaction remained light orange (Orange circles). 2. Ele: Agarose gel electrophoresis of LAMP products. Positive result (+). Negative result (−). Table S4. Comparison of analytical specificity and sensitivity of the LAMP assays and real-time PCR. [file 43008_2024_157_MOESM1_ESM.pdf]

Table S1 Fungal samples used for the LAMP assay development

**Pathogen**	**Host**	**Location**	**Samples No.**
Chrysomyxa diebuensis C.J.You & J.Cao	*Picea asperata* Mast.	Sichuan	10
Chrysomyxa ledi de Bary	*Picea jezoensis* Carr.	Jilin	10
Chrysomyxa pyrolae (DC.) Rostr	*Picea schrenkiana* Fischet Mey.	Xinjiang	10
Chrysomyxa qilianensis Y.C. Wang, X.B. Wu & B. Li	*Picea crassifolia* Kom.	Qinghai	10
Chrysomyxa qilianensis Y.C. Wang, X.B. Wu & B. Li	*Picea crassifolia* Kom.	Gansu	10
Chrysomyxa rhododendri de Bary	*Ledum palustre* L. var. *dilatatum* Wahlanberg	Heilongjiang	10
Chrysomyxa strumaria C. J. You & R. Wang	*Picea wilsonii* Mast.	Shaanxi	10
Chrysomyxa succinea (Sacc.) Tranzschel	*Picea wilsonii* Mast.	Shaanxi	10

Chrysomyxa woroninii Tranz (Wint)	*Picea crassifolia* Kom.	Qinghai	10
Chrysomyxa woroninii Tranz (Wint)	*Picea likiangensis var. rubescens* Rehder & E. H. Wilson	Sichuan	10
Chrysomyxa yunnanensis (P.E. Crane) C.J. You & C.M. Tian	*Picea likiangensis* (Franch.) Pritz. var. *linzhiensis* Cheng et L.K.Fu	Tibet	10
Chrysomyxa zhuoniensis C.J.You & J.Cao	*Rhododendron phaeochrysum* Balf. f. et W. W. Smith	Tibet	10
Coleosporium perillae Kom.	*Perilla frutescens* (L.) Britt.	Shanxi	5
Coleosporium pulsatilla Rupr.	*Pinus sylvestris* var. mongolica Litv	Inner Mongolia	5
Coleosporium salviae Dietel	*Salvia japonica* Thunb.	Tibet	5
Lophodermium piceae (Fuckel) Höhn.	*Picea crassifolia* Kom.	Qinghai	5
Lophodermium formosana Syd. & P. Syd.	*Deutzia scabra* Thunb.	Inner Mongolia	5

Pucciniastrum pyrolae (J.F. Gmel.) J. Schröt	*Pyrola calliantha* H. Andr.	Inner Mongolia	3
Pucciniastrum tiliae Miyabe	*Abies holophylla* Maxim.	Heilongjiang	3
Rhizosphaera kalkhoffii Bubák	*Picea crassifolia* Kom.	Xinjiang	3
Rhizosphaera kalkhoffii Bubák	*Picea crassifolia* Kom.	Gansu	3
Thekopsora areolata (Fr.) Magnus	*Picea schrenkiana* var.*tianschanica* (Rupr.) W.C.Cheng & S.H.Fu	Xinjiang	3
Uredinopsis kameiana Faull	*Abies fabri* (Mast.) Craib	Gansu	3

Table S2 Primers used for the detection of *Chrysomyxa* using LAMP assays

Primers	Label	The Sequence of the Primer 5'–3'
1	F3	AGGAGTGTGGTGCGTTAA
	B3	AGAGCCAGATTACAAATTTGG
	FIP	TCCCACCTGATTTGAGGTCTAAAAA-ACACTGCAGCCATTTGAC
	BIP	ACCCACTGAACTTAAGCATATCAA-TTTTCCCTCTTCACTCGC
2	F3	AGGAGTGTGGTGCGTTAA
	B3	AGAGCCAGATTACAAATTTGG
	FIP	TCCCACCTGATTTGAGGTCTAAAAA-CACTGCAGCCATTTGACT
	BIP	ACCCACTGAACTTAAGCATATCAA-TTTTCCCTCTTCACTCGC
3	F3	AGGAGTGTGGTGCGTTAA
	B3	AGAGCCAGATTACAAATTTGG
	FIP	TCCCACCTGATTTGAGGTCTAAAAA-CACTGCAGCCATTTGACTT
	BIP	ACCCACTGAACTTAAGCATATCAA-TTTTCCCTCTTCACTCGC
4	F3	AGGAGTGTGGTGCGTTAA
	B3	AGAGCCAGATTACAAATTTGG

	FIP	TCCCACCTGATTTGAGGTCTAAAAA-ACTGCAGCCATTTGACTT
	BIP	ACCCACTGAACTTAAGCATATCAA-TTTTCCCTCTTCACTCGC
5	F3	TGCGTTAAAAAACACTGCA
	B3	AGAGCCAGATTACAAATTTGG
	FIP	TCCCACCTGATTTGAGGTCTAAAAA-GCCATTTGACTTTTGATAGATAGC
	BIP	ACCCACTGAACTTAAGCATATCAA-TTTTCCCTCTTCACTCGC
6	F3	TGCGTTAAAAAACACTGCA
	B3	AGAGCCAGATTACAAATTTGG
	FIP	TCCCACCTGATTTGAGGTCTAAAAA-CCATTTGACTTTTGATAGATAGC
	BIP	ACCCACTGAACTTAAGCATATCAA-TTTTCCCTCTTCACTCGC
7	F3	TGCGTTAAAAAACACTGCA
	B3	AGAGCCAGATTACAAATTTGG
	FIP	TCCCACCTGATTTGAGGTCTAAAAA-CCATTTGACTTTTGATAGATAGCT
	BIP	ACCCACTGAACTTAAGCATATCAA-TTTTCCCTCTTCACTCGC

8	F3	TGCGTTAAAAAACACTGCA
	B3	CCCTGAAAAGAGCCAGAT
	FIP	TCCCACCTGATTTGAGGTCTAAAAA-ATTTGACTTTTGATAGATAGCTTCC
	BIP	AGTGGAGGAAAAGAAACTAACAAGG-AAATTTGGGCTTTTCCCTC
9	F3	TGCGTTAAAAAACACTGCA
	B3	CCCTGAAAAGAGCCAGAT
	FIP	TCCCACCTGATTTGAGGTCTAAAAA-TTTGACTTTTGATAGATAGCTTCC
	BIP	AGTGGAGGAAAAGAAACTAACAAGG-AAATTTGGGCTTTTCCCTC
10	F3	TGCGTTAAAAAACACTGCA
	B3	CCCTGAAAAGAGCCAGAT
	FIP	TCCCACCTGATTTGAGGTCTAAAAA-TTGACTTTTGATAGATAGCTTCC
	BIP	AGTGGAGGAAAAGAAACTAACAAGG-AAATTTGGGCTTTTCCCTC
11	F3	TCGATGAAGAACACAGTGAA
	B3	GTACTTATATATTTAAGGTGAGCCA
	FIP	TGCAAGGTGCGTTCAAAGATT-GTGATAAGTAATGTGAATTGCAGAA

	BIP	CTGTTTGAGTGTCATGAAACCCT-ATAACGGCAACACCCAAC
12	F3	AAGAACACAGTGAAATGTGAT
	B3	GTACTTATATATTTAAGGTGAGCCA
	FIP	ATCCAAAAGGTGCAAGGTGC-ATGTGAATTGCAGAATTCAGT
	BIP	CTGTTTGAGTGTCATGAAACCCT-ATAACGGCAACACCCAAC
13	F3	CAGTGAAATGTGATAAGTAATGTGA
	B3	GTACTTATATATTTAAGGTGAGCCA
	FIP	GTACCTTTCGGAATATCCAAAAGGT-CAGAATTCAGTGAATCATCGAATC
	BIP	CTGTTTGAGTGTCATGAAACCCT-ATAACGGCAACACCCAAC

Table S3 Detection of thirteen sets of *Chrysomyxa* genus-specific LAMP primers using visual LAMP and gel electrophoresis.

Pathogen	Primer / Result	1	2	3	4	5	6	7	8	9	10	11	12	13
Chrysomyxa qilianensis	Clr	🟢	🟢	🟢	🟢	🟢	🟠	🟢	🟢	🟢	🟢	🟢	🟢	🟢
	Ele	+	+	+	+	+	−	+	+	+	+	+	+	+
Chrysomyxa succinea	Clr	🟢	🟢	🟢	🟢	🟢	🟠	🟠	🟠	🟠	🟠	🟢	🟢	🟠
	Ele	+	+	+	+	+	−	−	−	−	−	+	+	−
Chrysomyxa pyrolae	Clr	🟢	🟢	🟢	🟠	🟢	🟠	🟠	🟢	🟢	🟠	🟢	🟢	🟢
	Ele	+	+	+	−	+	−	−	+	+	−	+	+	+
Chrysomyxa dieduensis	Clr	🟠	🟢	🟢	🟠	🟢	🟠	🟠	🟢	🟢	🟠	🟢	🟢	🟢
	Ele	−	+	+	−	+	−	−	+	+	−	+	+	+
Chrysomyxa yunnanensis	Clr	🟢	🟢	🟢	🟢	🟢	🟠	🟢	🟢	🟢	🟢	🟢	🟢	🟢
	Ele	+	+	+	+	+	−	+	+	+	+	+	+	+
Chrysomyxa zhuoniensis	Clr	🟠	🟢	🟢	🟢	🟢	🟠	🟠	🟢	🟢	🟢	🟢	🟢	🟠
	Ele	−	+	+	+	+	−	−	+	+	+	+	+	−
Chrysomyxa ledi	Clr	🟢	🟢	🟢	🟠	🟠	🟠	🟠	🟢	🟢	🟠	🟢	🟢	🟠
	Ele	+	+	+	−	−	−	−	+	+	−	+	+	−
Chrysomyxa rhododendri	Clr	🟢	🟢	🟢	🟢	🟢	🟠	🟠	🟠	🟠	🟠	🟠	🟢	🟢
	Ele	+	+	+	+	+	−	−	−	−	−	−	+	+
Chrysomyxa woroninii	Clr	🟠	🟢	🟢	🟠	🟠	🟠	🟢	🟠	🟠	🟠	🟢	🟢	🟠

	**Ele**	−	+	+	−	−	−	+	−	−	−	+	+	−
Chrysomyxa	**Clr**	🟢	🟢	🟢	🟢	🟢	🟠	🟠	🟠	🟠	🟠	🟢	🟢	🟢
strumaria	**Ele**	+	+	+	+	+	−	−	−	−	−	+	+	+

1.Clr: Visual inspection of the LAMP results. The positive reaction indicated by color change from light orange to light green (Green circles). The negative reaction remained light orange (Orange circles)

2.Ele: Agarose gel electrophoresis of LAMP products. Positive result (+). Negative result (−)

Table S4 Comparison of analytical specificity and sensitivity of the LAMP assays and real-time PCR.

			Visual LAMP	Real-time PCR
	**Targets**	No. samples	No. Positive	No. Positive
Specificity	*Chrysomyxa diebuensis*	10	10	10
	Chrysomyxa ledi	10	10	10
	Chrysomyxa pyrolae	10	10	10
	Chrysomyxa qilianensis	20	20	20
	Chrysomyxa rhododendri	10	10	10
	Chrysomyxa strumaria	10	10	10
	Chrysomyxa succinea	10	10	10
	Chrysomyxa woroninii	20	20	20
	Chrysomyxa yunnanensis	10	10	10
	Chrysomyxa zhuoniensis	10	10	10
	**Non targets**			
	Coleosporium perillae	5	0	0
	Coleosporium pulsatilla	5	0	0
	Coleosporium salviae	5	0	0
	Lophodermium piceae	5	0	0
	Lophodermium formosana	5	0	0
	Pucciniastrum pyrolae	3	0	0
	Pucciniastrum tiliae	3	0	0
	Rhizosphaera kalkhoffii	6	0	0
	Thekopsora areolata	3	0	0
	Uredinopsis kameiana	3	0	0
			**LAMP**	**Real-time PCR**
	**Concentration**	No. rep.	No. positive	No. positive
**Sensitivity**	5.2 $\times 10^{-1}$ ng/ul	3	3	3
	5.2 $\times 10^{-2}$ ng/ul	3	3	3

5.2×10^{-3} ng/ul	3	3	0
5.2×10^{-4} ng/ul	3	3	0
5.2×10^{-5} ng/ul	3	3	0
5.2×10^{-6} ng/ul	3	3	0
5.2×10^{-7} ng/ul	3	0	0
