# Supplementary material for: Development and validation of a rapid loop-mediated isothermal amplification assay for the detection of Chrysomyxa and characterization of Chrysomyxa woroninii overwintering on Picea in China
Source: IMA Fungus. 2024 Aug 7;15:23. doi: 10.1186/s43008-024-00157-6 (PMC11304928; doi:10.1186/s43008-024-00157-6)
Supplement: Supplementary file 2 — Additional file 2 Figure. S1. Detection of the optimal LAMP primers for Chrysomyxa species. A. Visual: green color indicated the detection of DNA fragments specific to Chrysomyxa species; orange color indicates the lack of detection. B: In real-time qPCR equipment: amplification signals above the threshold indicated the detection of Chrysomyxa DNA; no PCR amplicon was detected in negative control (NC, ddH2O). Figure. S2. Optimization of temperature and time of LAMP reaction. Figure. S3. Sensitivity of LAMP assays assessed by gel electrophoresis analysis (Concentration of diluted DNA template from 5.2 × 10^−1^ to 5.2 × 10^−7^ ng/μL.). Figure. S4. Sensitivity of Real-time PCR assay in detecting Chrysomyxa. Positive results for concentrations from 5.2 ng/μL to 5.2 × 10^−2^ng/μL. Negative results for 5.2 × 10^−3^ and negative control (ddH2O). [file 43008_2024_157_MOESM2_ESM.pdf]

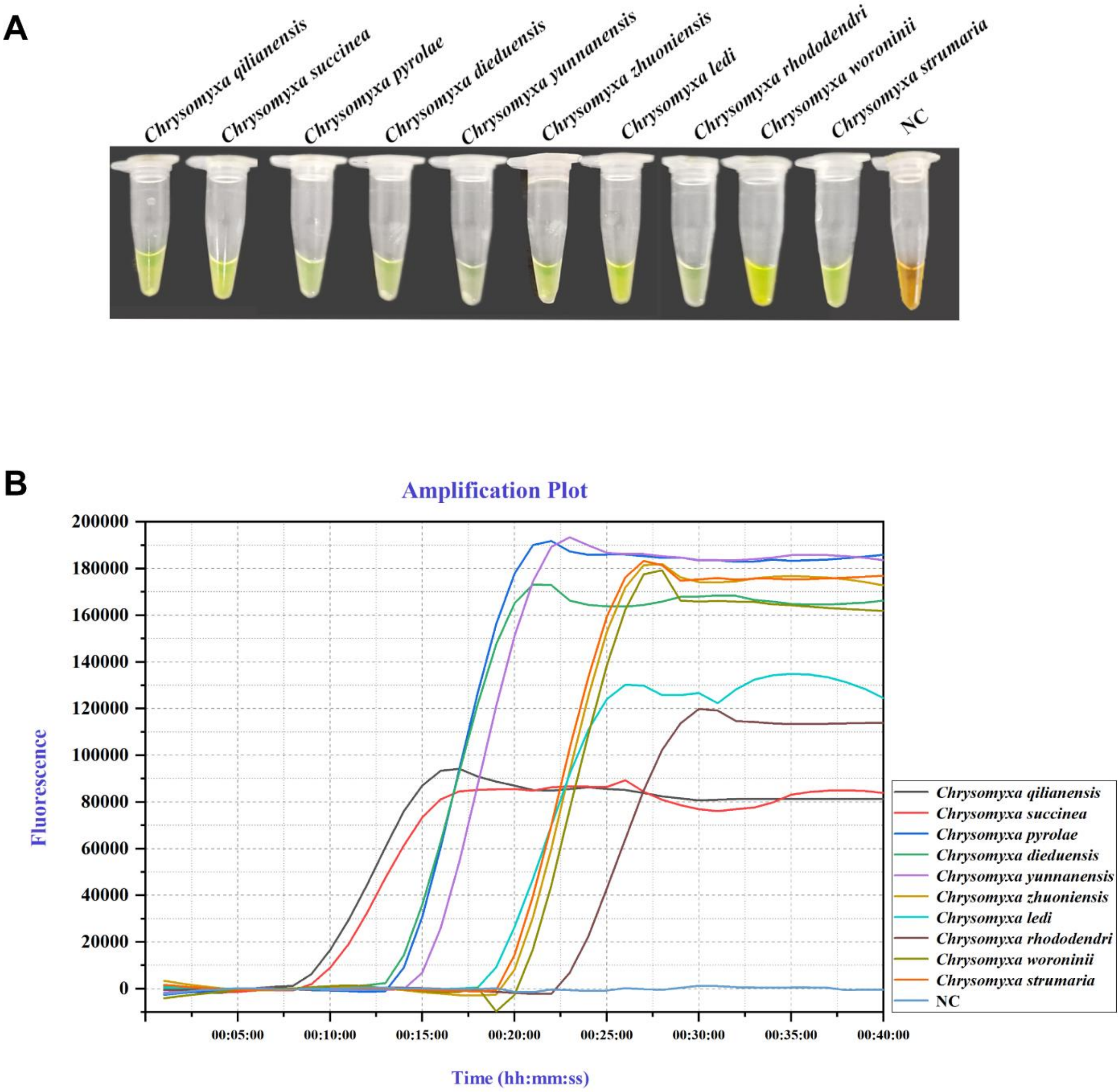

Figure. S1 Detection of the optimal LAMP primers for *Chrysomyxa* species.

A. Visual: green color indicated the detection of DNA fragments specific to *Chrysomyxa* species; orange color indicates the lack of detection. **B**: In real-time qPCR equipment: amplification signals above the threshold indicated the detection of *Chrysomyxa* DNA; no PCR amplicon was detected in negative control (NC, ddH2O).

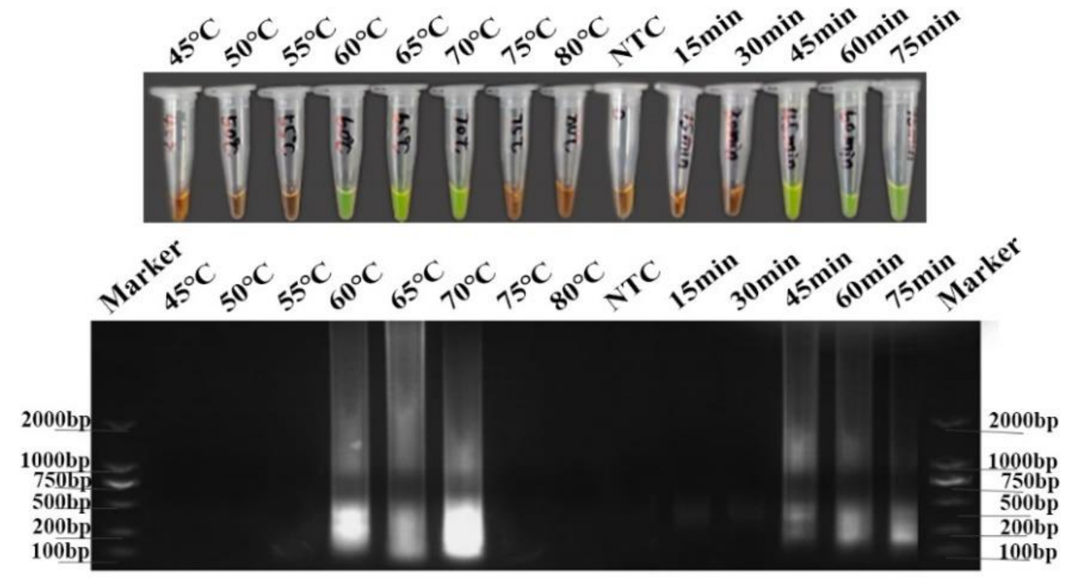

Figure S2. Optimization of temperature and time of LAMP reaction

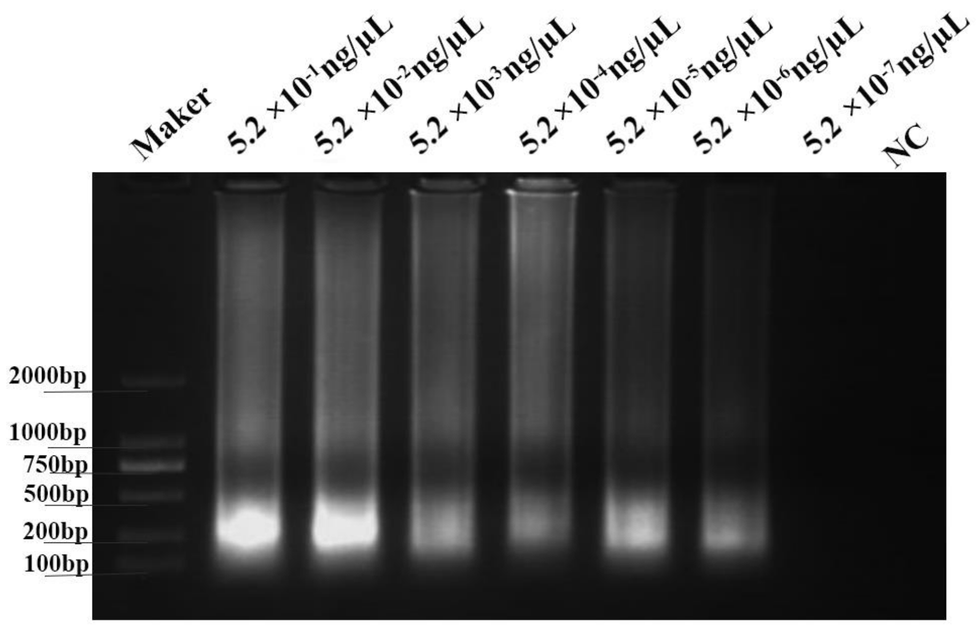

Figure. S3 Sensitivity of LAMP assays assessed by gel electrophoresis analysis

(Concentration of diluted DNA template from 5.2×10^{-1} to 5.2×10^{-7} ng/μL. NC negative control).

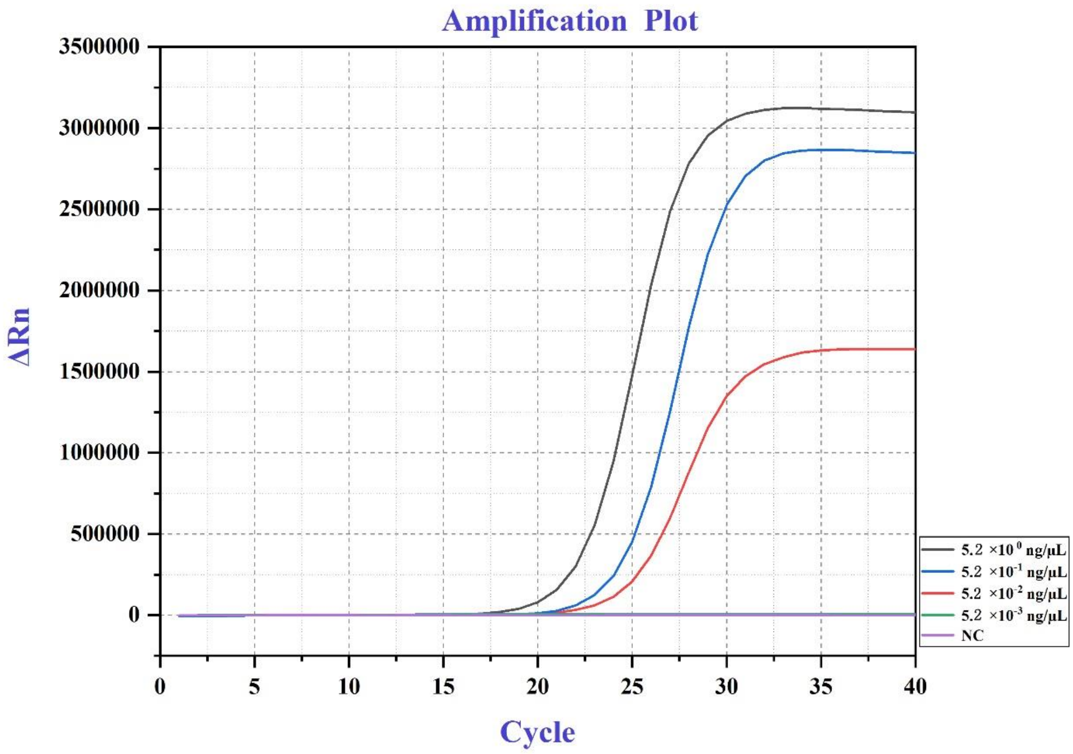

Figure. S4 Sensitivity of Real-time PCR assay in detecting *Chrysomyxa*. Positive results for concentrations from 5.2 ng/μL to 5.2×10^{-2}ng/μL. Negative results for 5.2×10^{-3} and negative control (ddH2O).
